# Assessing the effects of antidepressant use on stroke recurrence and related outcomes in ischemic stroke patients: a propensity score matched analysis

**DOI:** 10.3389/fphar.2025.1558703

**Published:** 2025-03-12

**Authors:** Faisal F. Alamri, Daniyah A. Almarghalani, Yasser Alatawi, Eman A. Alraddadi, Omar Babateen, Ahmed Alayyafi, Ziyad Almalki, Khaled Alsalhi, Khaled Alzahrani, Ahmed Alghamdi, Hussain Aldera, Vardan T. Karamyan

**Affiliations:** ^1^ Department of Basic Sciences, College of Science and Health Professions, King Saud bin Abdulaziz University for Health Sciences, Jeddah, Saudi Arabia; ^2^ King Abdullah International Medical Research Center, Jeddah, Saudi Arabia; ^3^ Department of Pharmacology and Toxicology, College of Pharmacy, Taif University, Taif, Saudi Arabia; ^4^ Stroke Research Unit, Taif University, Taif, Saudi Arabia; ^5^ Department of Pharmacy Practice, Faculty of Pharmacy, University of Tabuk, Tabuk, Saudi Arabia; ^6^ Department of Physiology, Faculty of Medicine, Umm Al-Qura University, Makkah, Saudi Arabia; ^7^ College of Medicine, King Saud Bin Abdulaziz University for Health Sciences, Jeddah, Saudi Arabia; ^8^ College of Medicine, King Saud Bin Abdulaziz University for Health Sciences, Riyadh, Saudi Arabia; ^9^ King Abdullah International Medical Research Center, Riyadh, Saudi Arabia; ^10^ Department of Foundational Medical Studies, Oakland University, Rochester, MI, United States; ^11^ Laboratory for Neurodegenerative Disease & Drug Discovery, William Beaumont School of Medicine, Oakland University, Rochester, MI, United States

**Keywords:** antidepressants, ischemic stroke, SSRI, stroke recurrence, stroke complications, mortality, propensity score matching (PSM)

## Abstract

The effect of antidepressant use, particularly that of selective serotonin reuptake inhibitors, on stroke outcomes remains unclear. This hospital-based, retrospective, observational study utilized propensity score-matching (PSM) to assess the association between antidepressant use, stroke-related outcomes, and complications. The study was conducted at King Abdulaziz Medical City (KAMC) in Jeddah and Riyadh and included 1,125 patients with acute-subacute ischemic stroke, of whom 1,025 were antidepressant non-users and 100 antidepressant users. After PSM, 200 patients (100 antidepressant users and 100 non-users) were included in the final analysis. This study aimed to assess the association between antidepressant use, stroke recurrence, and mortality. Additionally, the study examined the association between antidepressant use and stroke severity, functional independence, and incidence of post-stroke complications. The Kaplan-Meier analysis revealed no statistically significant differences in stroke recurrence (*p* = 0.5619) or mortality (*p* = 0.6433) between antidepressant users and non-users over the 1-year follow-up period. Additionally, no significant differences were observed in stroke severity at admission and discharge (*p* = 0.33210 and *p* = 0.78410, respectively) or functional independence (*p* = 0.9176 and *p* = 0.4383, respectively) between the two groups. These findings suggest that antidepressant use does not significantly affect stroke recurrence, mortality, stroke severity, or functional independence. However, further large-scale studies are warranted to validate these findings and investigate potential confounding factors, such as stroke subtypes, co-use of certain medications, and physical activity.

## 1 Introduction

Ischemic stroke is a major global health challenge, with a 70% increase in global stroke incidence over the past 3 decades, exceeding 12.2 million new cases annually (Feigin et al., 2021; Pu et al., 2023; Alraddadi et al., 2025a). In Saudi Arabia, the prevalence of stroke is estimated at 29 cases per 100,000 people, with ischemic stroke accounting for approximately 80% of cases (Alqahtani et al., 2020). Stroke can affect various brain functions, including motor and cognitive abilities, and may lead to complications, such as seizures and depression. For instance, one meta-analysis reported that one in six patients had depression before stroke, which was associated with an increased risk of post-stroke depression (PSD) (Taylor-Rowan et al., 2019). PSD affects nearly 30%–40% of stroke survivors (Hackett et al., 2005; Ayerbe et al., 2013). In addition to their direct effects on quality of life, depressive symptoms impair stroke recovery, worsen functional outcomes, and independently increase the risk of stroke recurrence and mortality (Ayerbe et al., 2013; Wu et al., 2019).

Selective serotonin reuptake inhibitors (SSRIs) are commonly used for the management of depression, effectively alleviating depressive symptoms in most patients within several weeks (Mortensen and Andersen, 2021). However, the direct effects on stroke severity, functional independence, complications, long-term stroke recurrence, and mortality remain controversial. SSRIs have been investigated for their potential to enhance overall stroke recovery. A systematic review and meta-analysis analyzed data from 44 studies involving 16,164 patients and found that SSRIs significantly reduced the incidence of depression and anxiety in post-stroke patients (Kalbouneh et al., 2022). Additionally, SSRI use has been associated with improvements in motor function, cognitive abilities, and dependence levels (Kalbouneh et al., 2022). Similarly, additional studies have revealed that SSRI use starting after the onset of stroke significantly improves motor and cognitive function post-stroke (Jorge et al., 2010; Chollet et al., 2011). The neuroprotective effects of SSRIs in stroke are thought to occur through various mechanisms, including promotion of neurogenesis, improvement of cerebral blood flow, modulation of inflammation (Lim et al., 2009), and enhancement of neurotransmitter function (Pinto et al., 2017). In the context of stroke recovery, SSRIs are deemed to facilitate neural repair and enhance brain plasticity, thereby supporting functional recovery and rehabilitation efforts (Chollet et al., 2011; Zahrai et al., 2020; Schneider et al., 2021; Alamri et al., 2021).

However, it must be noted that three recent multicenter clinical trials evaluating the effect of fluoxetine on post-stroke recovery (AFFINITY (Hankey et al., 2020), EFFECTS (Lundström et al., 2020), and FOCUS (Dennis et al., 2019) found no evidence of a positive effect of this SSRI on stroke recovery, although criticism followed regarding the study design and outcome measures that could have impacted the results of these studies (Ward and Carmichael, 2020; Kwakkel et al., 2020; Cramer, 2020; Karamyan, 2023). Furthermore, other studies reported an increased risk of stroke recurrence associated with SSRI use (Juang et al., 2015). In addition, an analysis of Danish registry data indicated that while SSRIs may reduce the risk of new cardiovascular events, they are also associated with an increased risk of bleeding (Mortensen et al., 2013). Moreover, two Cochrane systematic reviews concluded that SSRIs do not significantly enhance stroke recovery and are associated with an increased risk of hemorrhage (Mead et al., 2012; Legg et al., 2020). Furthermore, a meta-analysis revealed that even after adjusting for depression as a covariate, SSRI use remained an independent risk factor for stroke incidence (Trajkova et al., 2019). However, the influence of antidepressants on long-term stroke recurrence and mortality in patients with recurrent ischemic stroke has not yet been tested in Saudi Arabia.

Given the conflicting findings on the impact of SSRI use on stroke outcomes, this multicenter, hospital-based propensity score-matched (PSM) study aimed to investigate the association between antidepressant use, specifically SSRIs and serotonin-norepinephrine reuptake inhibitors (SNRIs), and clinical severity, recurrence, mortality, functional independence, and complications of ischemic stroke.

## 2 Materials and methods

### 2.1 Study design and setting

This hospital-based retrospective observational PSM study was conducted at King Abdulaziz Medical City (KAMC) in Riyadh and Jeddah, Saudi Arabia, between January 2016 and September 2022. Both hospitals are academic tertiary referral centers with a capacity of 509 beds in Jeddah and 690 beds in Riyadh.

### 2.2 Participants and data collection

**FIGURE 1:**
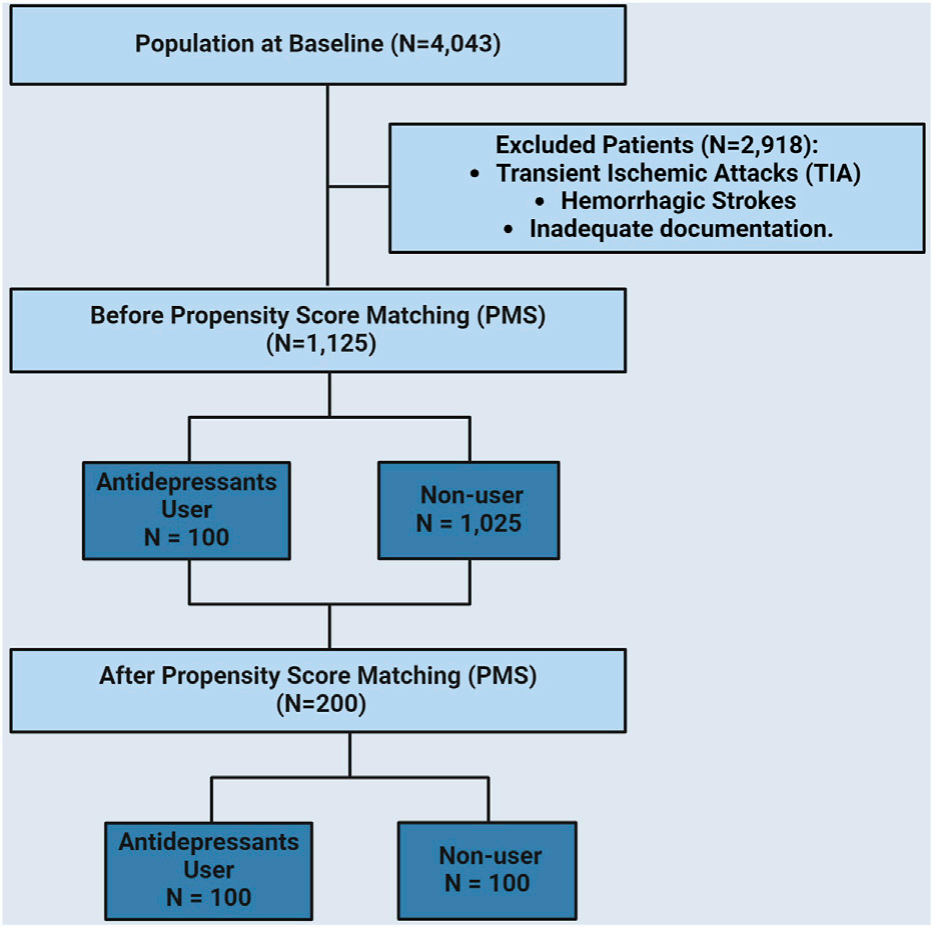
A flowchart of patients’ inclusion criteria.

Participants were required to meet specific inclusion criteria: they had to be adult patients aged 18 years or older and diagnosed with acute or subacute ischemic stroke. The exclusion criteria included transient ischemic attacks (TIA), hemorrhagic strokes, and inadequate documentation (Figure 1). Collected data were retrieved from the electronic medical records system (BEST-Care^®^ 2.0) and included patient demographics (gender and age) as well as clinical characteristics. Stroke severity and functional independence at admission and discharge after stroke were assessed using the National Institutes of Health Stroke Scale (NIHSS), whereas functional independence was evaluated using the modified Rankin Scale (mRS) (Alharbi et al., 2022; Alamri et al., 2024). The Trial of ORG 10172 in Acute Stroke Treatment (TOAST) classification of ischemic stroke was used to categorize ischemic stroke etiology into five subtypes: 1) large-artery atherosclerosis, 2) cardioembolism, 3) small-vessel occlusion, 4) stroke of other determined etiologies, and 5) stroke of undetermined etiology (Alraddadi et al., 2024). In addition, data on drug therapy, medical history of stroke, prior administration of tissue-type plasminogen activators (tPA), endovascular thrombectomy, and admission to the intensive care unit (ICU) were collected. Information on comorbid medical conditions, such as diabetes mellitus (DM), hypertension (HTN), atrial fibrillation (AF), dyslipidemia, depression, and dementia was also included. Furthermore, the signs, symptoms, and post-stroke complications have been documented, including motor dysfunction, dysarthria, aphasia, stroke-related pneumonia, deep venous thrombosis (DVT), pulmonary embolism (PE), cerebral edema, stroke recurrence, and mortality. Finally, data on the history of depression and use of antidepressant medications, including SSRIs (e.g., citalopram, fluoxetine, sertraline, paroxetine, and escitalopram) and SNRIs (e.g., venlafaxine), were also retrieved.

### 2.3 Exposure and outcome measures

The primary exposure variable in this study was antidepressant use (SSRIs or SNRIs) at any point during the past year or within the first 7 days following acute ischemic stroke. The primary endpoint was the incidence of stroke recurrence or mortality within 1 year of the index stroke (Alraddadi et al., 2025b). Neurological impairment, functional independence, and post-stroke complications were considered secondary endpoints.

### 2.4 Propensity score matching

To minimize the possibility of selection bias and maximize sample size, several precautionary measures were implemented: 1) The propensity score for antidepressant use was calculated for all patients, and 2) patients identified as antidepressant users were matched to non-users with similar propensity scores. For propensity score matching, we used demographic and clinical characteristics including gender, age, DM, HTN, dyslipidemia, prior stroke history, AF, and dementia. The objective of this study was to create two comparable patient groups: one exposed to antidepressants (SSRIs and SNRIs) and a matched control group of non-users. The nearest-neighbor matching approach was employed to achieve one-to-one matching without replacement using a caliper width of 0.2. (Austin, 2011; Mortensen et al., 2013). By utilizing a robust PSM design, this study minimized confounding factors and provided more precise insights into the potential benefits or risks associated with antidepressants in stroke patients (Wang, 2021).

### 2.5 Ethics statement

Ethical approval was obtained from the King Abdullah International Medical Research Center (KAIMRC) Institutional Review Board (SP22J/054/03). The requirement for informed consent was waived due to the retrospective nature of the study.

### 2.6 Statistical analysis

The baseline characteristics of the study sample before and after propensity score matching were summarized using a descriptive analysis. Mean ± standard deviation (SD) or median (interquartile range (IQR) was used to present continuous variables, while frequencies and percentages were used to present categorical variables. To compare groups, paired t-tests were conducted for continuous variables and chi-square tests were used for categorical variables. Conditional logistic regression analyses, both univariate and multivariate, were performed to investigate the relationship among antidepressant exposure, stroke symptoms, and complications, accounting for potential confounding variables when appropriate. Odds ratios (ORs) with 95% confidence intervals (CIs) were calculated to assess the degree of association. Additionally, survival analyses of the study outcomes (mortality and stroke recurrence) between those exposed and not exposed to antidepressants were performed using Kaplan-Meier curves and log-rank test. Cox proportional hazard regression was used to identify the factors associated with each study outcome. The model was adjusted for antidepressant use, and the variables showed significant differences between antidepressants after propensity score matching. Additionally, hazard ratios (HR) with corresponding 95% CIs were calculated to assess the magnitude of the associations. Statistical significance was set at *p* < 0.05. Statistical analyses were conducted using SAS software (version 9.4; SAS Institute, Inc., Cary, NC, United States).

## 3 Results

### 3.1 Baseline characteristics before and after propensity score matching

From January 2016 to September 2022, 4,043 admissions were recorded in the medical wards, with 1,125 patients fulfilling the inclusion criteria. Within this cohort, 1,025 participants were identified as non-users of antidepressants and 100 were classified as users. Males accounted for (62.05%) of the non-users group and (59%) of the users group. After applying PSM, the final analysis included 100 non-users and 100 users (Figure 1). Population characteristics are presented in Table 1; before PSM, the antidepressants users group had significantly higher rates of prior stroke (49% vs 37.95%, *p* = 0.0305), HTN (93% vs 80.86%, *p* = 0.0026), DM (82% vs 72.46%, *p* = 0.0396), AF (18% vs 9.77%, *p* = 0.0103), dementia (18% vs 5.37%, *p* < 0.0001), and depression (41% vs 8.42%, *p* < 0.0001) compared to non-users. No significant difference was observed between antidepressant users and non-users in dyslipidemia (53% vs 43.85%, *p* = 0.0789). After PSM, there were no significant differences in age, gender, stroke subtype, tPA use, mechanical thrombectomy, ICU admission, or history of comorbidities (HTN, DM, AF, dyslipidemia, or dementia) between the groups. However, depression remained significantly higher in the antidepressant user group (41% vs 9%, *p* < 0.0001).

**TABLE 1:** Basic characteristics.

Characteristics	Before PSM			After PSM		
	Users	Non-users	*p*-value	Users	Non-users	*p*-value
Age, mean ± SD	66.47 (11.90)	65.55 (12.90)	0.4929	66.47 (11.90)	66.14 (10.34)	0.8038
Male, n (%)	59 (59.00)	636 (62.05)	0.5493	55 (55.00)	59 (59.00)	0.5678
Stroke subtype, n (%)^a^						
1	48 (51.61)	467 (51.15)	0.3472	48 (51.61)	41 (46.59)	0.488
2	18 (19.35)	235 (25.74)		18 (19.35)	26 (29.55)	
3	5 (5.38)	59 (6.46)		5 (5.38)	6 (6.82)	
4	4 (4.30)	20 (2.19)		4 (4.30)	2 (2.27)	
5	18 (19.35)	132 (14.46)		18 (19.35)	13 (14.77)	
Unknown/missing	119 (11.00)			19 (9.5)		
Prior stroke, n (%)	49 (49.00)	389 (37.95)	0.0305	49 (49.00)	45 (45.00)	0.5709
tPA therapy, n (%)^a^	7 (7.00)	100 (9.78)	0.367	7 (7.00)	12 (12.00)	0.2279
Mechanical thrombectomy, n (%)	5 (5.00)	54 (5.28)	0.9051	5 (5.00)	6 (6.00)	0.7564
ICU admission, n (%)^a^	36 (36.73)	303 (29.68)	0.1464	36 (36.73)	25 (25.25)	0.0814
History of comorbidities, n (%)						
Hypertension, n (%)	93 (93.00)	828 (80.86)	0.0026	93 (93.00)	92 (92.00)	0.7883
Diabetes Mellitus, n (%)	82 (82.00)	742 (72.46)	0.0396	82 (82.00)	90 (90.00)	0.103
Dyslipidemia, n (%)	53 (53.00)	449 (43.85)	0.0789	53 (53.00)	59 (59.00)	0.3927
Atrial Fibrillation, n (%)	18 (18.00)	100 (9.77)	0.0103	18 (18.00)	13 (13.00)	0.3286
Dementia, n (%)	18 (18.00)	55 (5.37)	<0.0001	18 (18.00)	19 (19.00)	0.8555
Depression, n (%)^a^	41 (41.00)	86 (8.42)	<0.0001	41 (41.00)	9 (9.00)	**< 0.0001**
Aspirin, n (%)^a^	85 (85.00)	878 (85.66)	0.8579	85 (85.00)	94 (94.00)	**0.0379**
Clopidogrel, n (%)^a^	57 (57.00)	652 (63.62)	0.1912	57 (57.00)	70 (70.00)	0.0562

^a^ Variables not included in propensity score matching.

PSM, propensity score matching; SD, standard deviation; tPA, tissue plasminogen activator; ICU, intensive care unit.

### 3.2 Antidepressant exposure and stroke severity at admission and discharge after propensity score matching

At admission, the median NIHSS score was 8 (IQR: 5–11) in the antidepressant user group and 6 (IQR: 4–11) in the non-user group; however, the difference was not statistically significant (*p* = 0.3321). At discharge, the median NIHSS score was similar between the two groups (6.5 [IQR: 3–12] in users vs 6 [IQR: 2–13] in non-users, *p* = 0.7841). Similarly, there were no differences in the median mRS scores at admission (4 [IQR: 0–5] in users vs 4 [IQR: 1–5] in non-users, *p* = 0.9176) or at discharge (5 [IQR: 4–5] in users vs 4 [IQR: 2–5] in non-users, *p* = 0.4383; Table 2).

**TABLE 2:** Comparisons of stroke severity at admission and discharge between users and non-users.

Variable	User	Non-user	*p*-value
NIHSS at admission	8 (5–11)	6 (4–11)	0.3321
NIHSS at discharge	6.5 (3–12)	6 (2–13)	0.7841
mRS at admission	4 (0–5)	4 (1–5)	0.9176
mRS at discharge	5 (4–5)	4 (2–5)	0.4383

Data are shown as median (25th–75th percentiles).

NIHSS, national institutes of health stroke scale; mRS, modified Rankin Scale.

### 3.3 Assessment of antidepressant exposure, mortality, and stroke recurrence after propensity score matching

**FIGURE 2:**
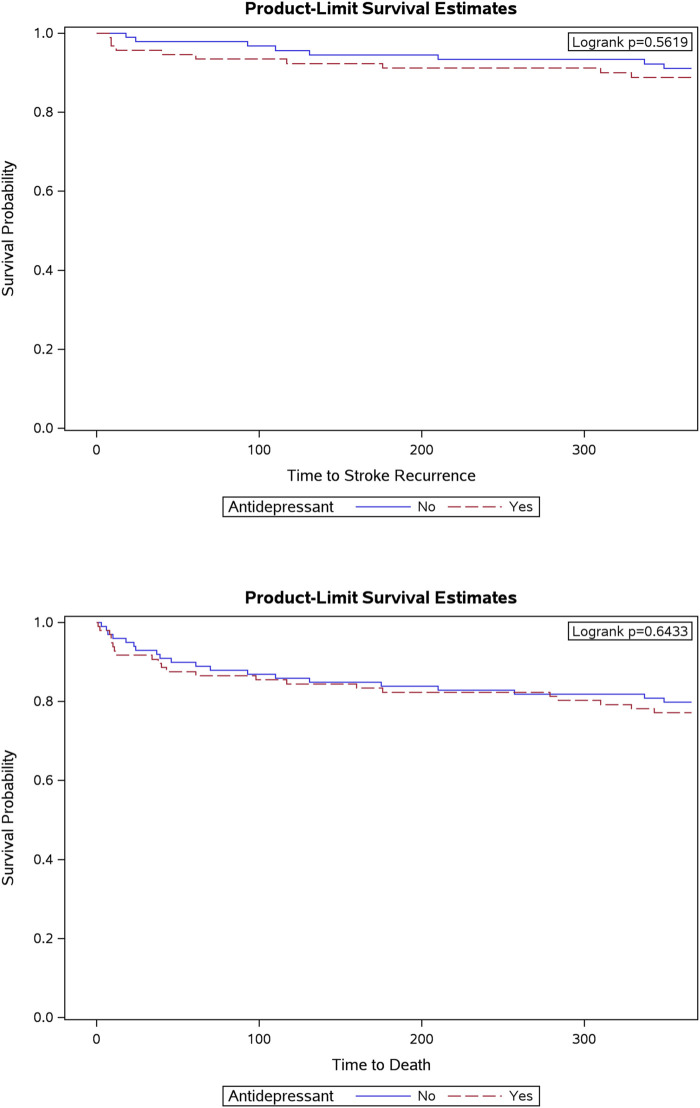
Kaplan-Meier curves by antidepressants use.

Kaplan–Meier analysis of stroke recurrence over time showed no differences in stroke recurrence rates between antidepressant users and non-users (*p* = 0.5619; Figure 2). Additionally, the log-rank test for mortality showed no statistically significant differences in survival probabilities over time between antidepressant users and non-users (*p* = 0.6433; Figure 2). Furthermore, Cox regression model results were used to assess whether antidepressant use was associated with any of the study outcomes, adjusting for confounding factors identified as significant after the propensity score matching (specifically, depression and aspirin). The results indicated no significant association between any of the variables and study outcomes. For example, there was no association between antidepressant use and aspirin exposure (HR = 1.36, 95% CI: 0.71–2.60, *p* = 0.3508), depression diagnosis (HR = 1.32, 95% CI: 0.50–2.98, *p* = 0.5292), and mortality (HR = 2.20, 95% CI: 0.91–5.12, *p* = 0.1052).

### 3.4 Stroke symptoms and complications after propensity score matching

Unadjusted conditional logistic regression analysis revealed no statistically significant association between antidepressant use and various stroke-related symptoms and complications, including upper and lower (right and left) limb motor impairment, aphasia, dysarthria, pneumonia, cerebral edema, and impaired consciousness. However, there were marginally increased odds of DVT-PE among the antidepressant user group, but the association was not statistically significant (OR = 2.50, 95% CI: 0.97–6.44, *p* = 0.0578; Table 3).

**TABLE 3:** Unadjusted association between antidepressant use and stroke symptoms and complications.

Variable	OR	95% CI	*p*-value*
LL Motor Impairment	1.50	0.85–2.64	0.1601
LR Motor Impairment	1.46	0.85–2.50	0.1761
UL Motor Impairment	1.46	0.87–2.45	0.1546
UR Motor Impairment	1.70	0.98–2.95	0.0597
Aphasia	1.19	0.67–2.13	0.5558
Dysarthria	1.19	0.67–2.13	0.5558
Pneumonia	1.24	0.65–2.34	0.5172
Cerebral edema	0.89	0.34–2.30	0.8085
DVT-PE	2.50	0.97–6.44	0.0578
Impaired consciousness	1.53	0.83–2.82	0.1731

*p-values were estimated using univariate paired conditional logistic regression.

R LL, lower left; L, lower right; UL, upper left; UR, upper right; DVT-PE, deep vein thrombosis–pulmonary embolism.

## 4 Discussion

In this study, the association between antidepressant use (SSRIs and SNRIs) and 1-year stroke recurrence and related outcomes in patients with acute subacute ischemic stroke was analyzed. No statistically significant differences were found in stroke severity, recurrence, mortality, complications, or functional independence between the user and non-user groups (Figure 2). These findings are consistent with earlier studies that found no significant differences in mortality in stroke patients treated with antidepressants (Dennis et al., 2019; Lundström et al., 2020; Hankey et al., 2020). However, another study revealed an increase in mortality among SSRIs users, potentially due to a higher risk of bleeding (Mortensen et al., 2013). That report raised further concerns regarding the safety profile of antidepressants, particularly for vulnerable populations such as individuals with a history of stroke or other cardiovascular conditions (Mortensen et al., 2013). Nevertheless, further studies are required to confirm whether SSRI use is associated with a higher risk of mortality in patients with stroke.

The current study found a small, albeit non-significant, increase in the rate of stroke recurrence within 1 year among antidepressant users (Figure 2). This trend aligns with the previous findings. Notably, Juang et al. identified a potential association between antidepressant use and an increased risk of recurrent stroke among Taiwanese patients (Juang et al., 2015). Moreover, a systematic review and meta-analysis indicated that, even after adjusting for depression, antidepressant use was independently associated with a higher risk of stroke (Trajkova et al., 2019). Understanding the mechanisms underlying this trend, including its potential impact on adherence to secondary prevention strategies, is crucial for optimizing patient care. Future research with a larger sample size is needed to clarify the relationship between antidepressant use and stroke recurrence, and to identify subpopulations that may be more vulnerable to these risks. Drug combinations (e.g., antithrombotics) and stroke subtypes may be especially important in answering this question.

The current study found no significant differences between antidepressant users and non-users in stroke severity, as measured by the NIHSS, or in functional independence, as measured by the mRS, at both admission and discharge (Table 2). These findings are consistent with those of a previous study that reported no significant increase in hospital stay duration or NIHSS scores among patients with ischemic stroke using SSRIs (Etherton et al., 2018). Similarly, two Cochrane reviews found that SSRIs did not significantly affect disability or functional independence (Mead et al., 2012; Legg et al., 2020). In contrast, a recent systematic review and meta-analysis reported that SSRI use in patients with stroke was associated with improvements in depression and anxiety symptoms, cognition, motor skills, and functional independence (Kalbouneh et al., 2022). Moreover, another study found that while SSRIs do not enhance functional independence, they are associated with improved survival rates in patients with ischemic stroke, suggesting potential long-term benefits beyond functional recovery (Heiberger et al., 2019). Interestingly, a randomized clinical trial involving patients with PSD found that compared to the placebo group, those receiving citalopram had significantly lower rates of stroke recurrence (Ece Çetin et al., 2022). This finding underscores the importance of treating PSD as it has been linked to impaired brain repair and hindered functional recovery after stroke (Nannetti et al., 2005; Matsuzaki et al., 2015). PSD affects stroke outcomes through multiple mechanisms, including excitotoxicity, increased levels of pro-inflammatory cytokines, and downregulation of brain-derived neurotrophic factor (BDNF), a key mediator of brain repair (Wijeratne and Sales, 2021). Nevertheless, discrepancies among antidepressant studies are mainly attributed to variations in sample sizes, study designs, patient populations, specific medications used, and their regimens. Given these inconsistencies, further well-designed placebo-controlled clinical trials are needed to better understand the impact of antidepressant therapy on stroke recurrence and recovery.

In the present study, unadjusted analysis revealed no statistically significant differences between antidepressant users and non-users in terms of stroke-related symptoms and complications, including motor impairment, aphasia, dysarthria, pneumonia, cerebral edema, and impaired consciousness (Table 3). However, a borderline increase in the odds of DVT-PE was observed among antidepressant users, which warrants further investigation. Certain classes of antidepressants, particularly SSRIs, are associated with an increased risk of DVT-PE (Wu et al., 2013; Adelborg et al., 2017). This relationship may stem from the effects of antidepressants on platelet function and coagulation pathways (Ishioka et al., 2015; Letmaier et al., 2018), although the mechanisms are not entirely clear because SSRIs are known to have profibrinolytic and antiplatelet properties (Andrade et al., 2010; Hoirisch-Clapauch and Nardi, 2019). Physicians should be aware of this potential risk when prescribing antidepressants to patients with stroke, particularly those with limited mobility, as immobilization is a significant risk factor for DVT-PE and an impediment to stroke recovery. Moreover, the current study did not find a statistically significant difference in stroke subtypes between groups. It is important to note that a limited number of studies investigated the association of antidepressant medication use and stroke outcomes in different stroke subtypes (Alqdwah-Fattouh et al., 2022; Broman et al., 2022). One study found that the use of SSRIs was not linked to a higher risk of non-cardioembolic ischemic stroke (Alqdwah-Fattouh et al., 2022). They found that SSRIs seemed to offer more pronounced protective effects than other antidepressants. In comparison, because of the small sample size of stroke subtypes, the current study did not further investigate the association between stroke subtypes and outcomes. Hence, considering the stroke subtype in such studies is essential to provide insight into which subpopulations would benefit from or at higher levels of antidepressants (Legg et al., 2019; Alqdwah-Fattouh et al., 2022; Broman et al., 2022).

In rehabilitation settings, post-stroke SSRI use is associated with improvements in motor and cognitive function recovery (Chollet et al., 2011; Mikami et al., 2011; Ng et al., 2015; Alamri et al., 2021). For instance, a small clinical trial demonstrated that the early administration of fluoxetine in combination with physiotherapy significantly enhanced motor recovery in patients with ischemic stroke with moderate to severe motor deficits (Chollet et al., 2011). These findings suggest that pharmacological modulation of innate brain plasticity may offer a promising therapeutic strategy for individuals with substantial motor impairment following ischemic stroke. The combination of drug therapy and rehabilitation appears to be key for promoting recovery (Ng et al., 2015; Alamri et al., 2021; Karamyan, 2023). Additional clinical trials are required to confirm the efficacy of this combined approach (Mikami et al., 2011; Ng et al., 2015; Alamri et al., 2021). The potential mechanisms underlying the beneficial effects of SSRIs on motor recovery include increased cortical excitability and reorganization, upregulation of neural growth, and angiogenic factors, all of which contribute to neuroplasticity, enhanced neurogenesis, and synaptic connectivity within the affected brain regions (Elzib et al., 2019; Szelenberger et al., 2020; Schneider et al., 2021). Their positive impact on stroke recovery and rehabilitation outcomes may be partially attributed to their indirect effects on mood and mental health (Nannetti et al., 2005). However, in non-rehabilitation settings, large multicenter trials of fluoxetine for stroke recovery (AFFINITY, FOCUS, and EFFECTS) have failed to demonstrate significant functional improvements (Dennis et al., 2019; Hankey et al., 2020; Lundström et al., 2020). Nevertheless, the design of these studies, such as the use of the mRS and the lack of paired rehabilitation, has been a subject of criticism from experts in the field (Ward and Carmichael, 2020; Kwakkel et al., 2020; Cramer, 2020; Karamyan, 2023).

Despite the beneficial mechanisms of serotonergic activation in stroke recovery, the inconsistencies among studies can be largely attributed to the complex role of serotonin in modulating various brain functions. Serotonin acts as an arterial vasoconstrictor that regulates the cerebral vasomotor tone (Bak et al., 2002; Mortensen et al., 2013; Maroteaux and Kilic, 2019). Additionally, it interferes with platelet aggregation and coagulation factors, potentially increasing the risk of bleeding or decreasing the recurrence of thrombosis and subsequent stroke (Bak et al., 2002; Mortensen et al., 2013; Maroteaux and Kilic, 2019). Moreover, potential interactions between fluoxetine and antiplatelet or anticoagulant medications have been suggested (Rahman et al., 2024). However, large clinical trials have demonstrated that fluoxetine does not significantly increase the risk of gastrointestinal or intracranial bleeding compared to placebo (Dennis et al., 2019; Hankey et al., 2020). The findings of this report are consistent with those of major stroke trials, indicating that the use of antidepressants did not significantly increase the risk of stroke recurrence. Nevertheless, safety concerns regarding the use of fluoxetine have been reported in large trials, including hyponatremia, bone fractures, risk of falls, and seizures (Dennis et al., 2019; Hankey et al., 2020; Lundström et al., 2020). Future studies are needed to determine which antidepressants are safer and more effective and to identify the patient populations that would benefit the most.

This study had several limitations. First, because this retrospective analysis was conducted at two centers in Saudi Arabia, the relatively small sample size may limit the generalizability of the findings to broader patient populations across different countries and healthcare settings. The retrospective design inherently introduces potential biases owing to its reliance on pre-existing medical records, which may contain incomplete data on confounding variables. Second, the study did not assess drug adherence, safety profiles, the effects of a specific drug, dosage, or duration of antidepressant therapy. These factors could have significant implications for ischemic stroke outcomes and should be explored in future studies. Furthermore, not all potential confounding variables were accounted for, including rehabilitation interventions and the use of anticoagulants and non-steroidal anti-inflammatory drugs, which are commonly prescribed to stroke patients and are known to increase bleeding risk when combined with antidepressants. Third, the follow-up period available for stroke recurrence analysis was limited, restricting the assessment of the long-term impact of antidepressant use on stroke outcomes. Future studies with extended follow-up periods will provide a comprehensive understanding of these effects. Moreover, although PSM was employed to control for confounding factors, this method has limitations. PSM cannot be fully adjusted for unmeasured confounders or capture the complexities of individual patient profiles. To address these limitations, future research should focus on prospective, large-scale, multicenter studies to validate the impact of antidepressant use on stroke outcomes. Additionally, further research is needed to consider combination therapy (e.g., rehabilitation) and to identify the optimal treatment regimen, study design, and specific patient populations that may derive the greatest benefit from antidepressant therapy in stroke care (Karamyan, 2023). Moreover, the current literature lacks sufficient data to investigate the association between antidepressant use and stroke outcomes across different stroke subtypes (Legg et al., 2019; Alqdwah-Fattouh et al., 2022). Understanding these variations is critical because stroke pathophysiology differs between stroke subtypes, which may influence the effect of antidepressants on recovery. Considering stroke subtypes in future research is essential to determine which subpopulations are most likely to benefit from antidepressants and which may be at a higher risk of adverse effects (Legg et al., 2019; Alqdwah-Fattouh et al., 2022; Broman et al., 2022).

### 4.1 Conclusion

These findings suggest that while antidepressant users had a higher prevalence of comorbidities at baseline, the use of antidepressants in stroke patients did not significantly affect stroke severity, functional outcomes, mortality, or stroke recurrence. These observations in Saudi Arabian patients are consistent with those in other populations. These findings highlight the necessity to carefully weigh the potential benefits and risks of antidepressants in patients with stroke. Further research is necessary to better understand the complex interplay between post-stroke depression and antidepressant use and their implications on stroke-related outcomes to improve the care of this vulnerable group of patients.

## Data Availability

The raw data supporting the conclusions of this article will be made available by the authors, without undue reservation.
